# Women’s Experiences with Nicotine and Cannabis Vaping During Pregnancy and Postpartum

**DOI:** 10.3390/healthcare13030223

**Published:** 2025-01-22

**Authors:** Andreea C. Brabete, Lorraine Greaves, Nancy Poole, Ella Huber, Julie Stinson

**Affiliations:** 1 Centre of Excellence for Women’s Health, B225-4500 Oak St., Vancouver, BC V6H 3N1, Canada; lgreaves@cw.bc.ca (L.G.); npoole@cw.bc.ca (N.P.); ellahuber95@gmail.com (E.H.); juliestinson7@gmail.com (J.S.); 2 School of Population and Public Health, Faculty of Medicine, University of British Columbia, Vancouver, BC V6T 1Z3, Canada

**Keywords:** vaping, nicotine, cannabis, pregnancy, postpartum

## Abstract

**Background/Objectives**: There is limited research on vaping during pregnancy and the postpartum period. Amid the legalization of cannabis in Canada, and evolving patterns of nicotine use, there is a growing need to understand how women experience using nicotine and cannabis vaping during pregnancy and postpartum. This information is essential to inform both women and healthcare providers (HCPs) and to develop resources and best practices for supporting women and healthcare services. **Methods**: In this descriptive study, a sample of 111 women who vaped nicotine and/or cannabis during pregnancy/postpartum was recruited via social media to answer survey questions on reasons for vaping, perceptions of the risks to fetal and maternal health, attitudes toward vaping, and reasons for consulting HCPs regarding vaping during pregnancy. **Results**: Among the 111 women, 51.4% vaped nicotine, 27.9% vaped cannabis, and 20.7% vaped both. Of the respondents, 63.1% were currently pregnant, while 36.9% were postpartum. Most participants (64.9%) reported vaping daily, followed by 15.3% with an inconsistent pattern, 9.9% vaping 1–2 days a week, and 9% vaping 5–6 days a week. Flavor preferences were prevalent, with fruit flavors being the most popular, followed by menthol/mint and candy, dessert, or sweet flavors. The primary reasons for vaping were relaxation, managing anxiety/depression, enjoyment, and the belief that vaping is less harmful than smoking. Women commonly consulted HCPs about potential harm to their pregnancy, fetal health, and their child’s health. **Conclusions**: The findings suggest that vaping among pregnant and postpartum women, particularly cannabis vaping, is perceived as healthier than smoking and is often used to manage mental and physical symptoms. These findings were used to create knowledge products to help guide HCPs’ conversations with women and provide evidence-based information on vaping.

## 1. Introduction

The term “vaping” refers to the act of inhaling and exhaling an aerosol produced by a vaping product. This includes aerosols from both nicotine and cannabis products, although the devices used for each may differ [1]. The perception that vaping nicotine is healthier than smoking stems from the absence of many of the harmful chemicals found in traditional cigarettes [2]. However, vaping either nicotine and/or cannabis is not without risks; the long-term health effects of vaping are still being studied, and concerns about health effects, mental health and addiction persist. While vaping nicotine may be a safer alternative for those trying to reduce smoking, it is not risk-free [2,3].

Nicotine and cannabis vaping reflect differing trends and patterns, dependent upon age, regulatory environments, prevailing practices and policies, common perceptions, and available evidence. Nicotine vaping rates among young adults aged 18–24 are notably higher than those among older age groups [4,5]. According to the 2022 Canadian Tobacco and Nicotine Survey (CNTS), the prevalence of ever vaping was 30.0% among youth aged 15 to 19 years, 47.5% among young adults aged 20 to 24 years, and 14.7% among adults aged 25 years and older [5]. The prevalence of cannabis vaping has also seen a significant increase in recent years [6]. Despite these trends, cannabis vaping remains less common than nicotine vaping across age groups [6]. The 2022 CNTS showed that the prevalence of ever vaping cannabis was 13.9% among youth aged 15 to 19 years, 28.7% among young adults aged 20 to 24 years, and 10.7% among adults aged 25 years and older [5]. These patterns are influenced by regulatory policies, with cannabis vaping being most prevalent in the USA, followed by Canada and England [7,8,9].

Similar patterns are observed among pregnant and postpartum women. While the prevalence of nicotine vaping is reported to range from 1.2% to 7% according to systematic reviews, it remains a significant concern for public health [10]. These studies indicate that pregnant women vape nicotine to reduce or quit smoking [10]. According to the Canadian Cannabis Survey, since 2018, the majority of women who had given birth in the past five years did not use cannabis during their last pregnancy or while breastfeeding [11]. The percentage reporting cannabis use during pregnancy or breastfeeding has remained under 10% since 2018 [11].

The most common reason reported by women for vaping nicotine in pregnancy is to stop smoking or to prevent a return to smoking and reduce harm to themselves, their fetuses, babies and others around them [10]. Other motivations for vaping included the ability to use e-cigarettes in smoke-free areas, curiosity, the lower cost compared to cigarettes, the familiar hand-to-mouth action similar to smoking, and the variety of available flavors [10]. On the contrary, pregnant women have reported using cannabis during pregnancy for its perceived therapeutic effects, uncertainty about adverse perinatal consequences, and lower costs compared to cigarettes [12]. A qualitative study found that while women held contradictory beliefs about continued use and expressed concerns about potential risks, they viewed cannabis as more natural and safer than tobacco, alcohol, other recreational drugs, and prescribed medicines [13]. Women with mental health issues, particularly depression, were more likely to use cannabis during pregnancy [14]. In another qualitative study, women reported using cannabis for reasons such as sensation-seeking for fun and enjoyment; symptom management of chronic conditions and conditions related to pregnancy; and coping with the unpleasant, but non-pathologized, experiences of life [15]. In this study, it was found that reasons for use shifted during pregnancy primarily to symptom management and during lactation, resembled those expressed before pregnancy [15].

The most recent systematic reviews on the health impacts of vaping nicotine during pregnancy highlight the limited, mixed and inconclusive state of the findings [16,17]. For example, a recent systematic review that includes evidence up to 2024 analyzed 26 studies [17]. This review examined the health impacts of nicotine vaping among pregnant and postpartum women, as well as their fetuses and infants, and compared exclusive vaping with exclusive smoking and non-use of nicotine or tobacco. Their findings indicated that current evidence is of poor quality, inconclusive and partial, posing challenges for women and clinicians. Compared to non-use, the majority of studies included in the review reported no increased risk of exclusive vaping (6 studies reported increased risk, 24 found no increased risk). Most of the included studies found similar health risks when comparing exclusive vaping to exclusive smoking [17]. Dual use of vaping and smoking was found to be associated with higher risks for key fetal and infant outcomes compared to exclusive vaping and non-use and was comparable to the risks associated with exclusive smoking. However, there is a notable lack of research on the effects of dual use, especially on maternal health outcomes [17]. The findings of this review exemplify the complex, mixed and inconclusive evidence that women and HCPs have to contend with when making decisions during pregnancy.

To date, no studies have examined whether different methods of cannabis use (such as smoking, vaping, sublingual administration, or ingestion) have distinct effects on fetal outcomes. While the specific effects of vaping cannabis during pregnancy remain largely unknown, vaping has become the second most common method of cannabis use among pregnant women [18]. One well-documented risk of prenatal cannabis exposure is low birth weight, with the risk increasing with heavier use [19,20,21,22,23]. Some research indicates that cannabis use during pregnancy may be linked to a higher incidence of placental abruption [24], and that there is also an association with preeclampsia and gestational diabetes [25]. There is also some evidence that cannabis use could lead to adverse growth outcomes and birth complications, though these findings remain inconclusive [26].

Current advice from Canadian health organizations is to avoid cannabis use during pregnancy and breastfeeding [27] in part due to potential risks to fetal and infant health, including low birth weight and preterm birth [28]. Women’s perceptions of risk of substance use during pregnancy are impacted by a combination of their knowledge, values, and social contexts [29]. Women’s risk perceptions are influenced by broader sociocultural, personal, and informational factors, underscoring the complexity and individuality of each woman’s risk interpretation [30]. They often compare the information received from HCPs with friends’ experiences and also their own experiences [29] and they self-justify their substance use [31]. Although media reports, health campaigns, and healthcare advice about risk are prevalent, conflicting recommendations also exist—sometimes offering very different perspectives [29]. Additionally, considering that women are aware of the risks, risk education interventions may not be very effective, as women tend to offer counterarguments that justify their actions [32]. For example, women perceive e-cigarettes to be safer in pregnancy than cigarette smoking [33].

Considering the scarcity of studies on nicotine and cannabis vaping, the aim of this study was to examine vaping patterns during pregnancy and postpartum, reasons for vaping and to assess women’s attitudes and perceptions regarding vaping, risks and fetal and maternal health during pregnancy, and reasons for consulting a healthcare provider regarding their vaping habits.

## 2. Materials and Methods

### 2.1. Participants and Procedure

Study participants were women who vaped nicotine and/or cannabis during pregnancy and postpartum. These women were recruited between October 2020 and September 2021 through the Centre of Excellence for Women’s Health Facebook, Twitter, and Instagram accounts. We also connected with partners such as the Society of Obstetricians and Gynaecologists of Canada and BC Association of Pregnancy Outreach Programs to post original recruitment content on their social media channels by providing a recruitment package that included messaging, graphics, and direct links to the survey. The procedures used in the study were approved by the UBC C&W Research Ethics Board (H20-03197). All participants provided written informed consent.

### 2.2. Measures

The questions for this survey were set up in Hosted in Canada Surveys, a Canadian survey platform used to collect encrypted data and safely hosted in Canada. We collected quantitative data on vaping experiences leading up to, during pregnancy and immediately postpartum. Participants were asked about their patterns of use, reasons for vaping, context of use, knowledge of health effects and relational and social influences. See Supplementary Table S1 for the questions.

### 2.3. Statistical Analysis

We used descriptive statistics to characterize the sample’s demographic characteristics. Considering the scarcity of studies that analyze vaping-related behaviours in women and more specifically in pregnant and postpartum women, we analyzed the frequency of user question categories and the stated reasons for vaping use, knowledge of health effects, and beliefs about vaping. All analyses were conducted using SPSS 29.

## 3. Results

A total of 137 respondents consented to participate. In total, 112 respondents reported vaping at least once, and of those, 57 vaped nicotine, 31 vaped cannabis, and 23 vaped both nicotine and cannabis. In addition, 1 participant was ineligible for the study as they noted they vape(d) herbs or blends of essential oil, leaving 111 eligible respondents who completed the survey. Among the sample, 51.4% vaped nicotine, 27.9% vaped cannabis, and 20.7% vaped cannabis and nicotine.

The ages of the respondents were between 20–25 (*n* = 49, 44.1%), followed by 26–30 (*n* = 22, 19.8%), and 31–35 (*n* = 21, 18.9%). Only one respondent was over the age of 40 (aged 42). The mean age was 25.9 years (*SD* = 5.68), and the mean number of children for women was 1.0 (*SD* = 0.92).

Among participants, 28.8% completed high school as their highest education level and 30.6% reported a household income of CAD 30,000 to CAD 44,999. Additionally, 46.8% identified their marital status as common law, and 57% had one child. In terms of demographics, 7.2% were First Nations, Métis or Inuk (Inuit); 75.7% identified as White, and 62% as heterosexual or straight (See Table 1).

Of all the respondents in the sample, 63.1% indicated that they were currently pregnant, while 36.9% indicated that they were currently postpartum (having delivered a baby less than two years ago). Among all pregnant and postpartum women who reported cannabis and both cannabis and nicotine, 50% reported vaping THC, 42.6% reported both THC and CBD, and 3.7% reported CBD and do not know, respectively.

More than half of the participants (64.9%) reported they vaped every day, followed by 15.3% that did not have a consistent pattern, 9.9% reported vaping 1–2 days a week and 9% reported 5–6 days per week. Half of the women (50.4%) answered that they enjoy vaping either “extremely” or “very much” followed by “moderately” (28.8%) and “slightly” (13.5%). Only 7.2% of the women reported “not at all”. Most of the participants (82.9%) reported that they bought their own vaping device, 8.1% obtained it from a friend, 5.4% from their partner, 1.8% from a relative, and 0.9% from a free sample and other reasons, respectively.

Participants who vaped nicotine and both cannabis and nicotine (*n* = 80) were asked what e-liquid flavours they have used. Fruit flavour (86.25%) was the most common e-liquid flavour followed by menthol or mint (45%) and candy, desserts and sweets (37.5%). The remaining options were selected by less than 10% of the participants and only 2.5% of the participants reported they do not use e-liquid.

Regarding substance use before pregnancy, 84.7% of the women answered that they drank alcohol before pregnancy (15.3% of the women answered they did not drink alcohol before pregnancy), 71.2% used cannabis (28.8% of the women answered they did not use cannabis before pregnancy) and 58.6% smoked tobacco (41.4% of the women reported they did not smoke tobacco before pregnancy). Among those who had used cannabis prior to pregnancy, participants reported they had vaped (57.0%) smoked (53.2%) and used edibles (21.5%).

### 3.1. Reasons for Vaping

Most frequently, women reported they vaped (nicotine and/or cannabis) to relax (92.8%), followed by vaping to help manage anxiety/depression during pregnancy/postpartum (85.6%), enjoyment of vaping (76.6%), and because vaping is less harmful than smoking (75.7%), as shown in Table 2.

When asked about vaping to improve or manage mental health or substance use concerns, vaping to improve or manage anxiety (82.9%) was most frequently reported, followed by depression and post-traumatic stress disorder or traumatic event, as shown in Table 3.

When asked about vaping to improve or manage physical symptoms, managing problems sleeping was most frequently reported (44.1%), followed by headaches/migraines (41.4%), lack of appetite (36.9%), nausea/vomiting (29.7%) and pain (28.8%), as shown in Table 4.

### 3.2. Perception of Health Effects

Among women who reported cannabis vaping, 80.6% reported perceiving low harm from cannabis vaping for their health, while 19.4% reported perceiving moderate harm, and none reported high harm. Among women who reported vaping nicotine, 36.8% reported perceiving low harm, 34.8% reported perceiving moderate harm, and 8.8% reported perceiving high harm. Among those who reported vaping both cannabis and nicotine when questioned about cannabis, 65.2% reported perceiving low harm, 34.8% reported perceiving moderate harm, and none reported perceiving high harm. When this same group of women was asked about nicotine, 39.1% reported perceiving low harm, 39.1% reported perceiving moderate harm, and 21.7% reported perceiving high harm.

Among women who were pregnant and reported vaping cannabis, 87% reported perceiving low harm for the fetus, 8.7% reported moderate harm and 4.3% reported high harm. Among women who were pregnant and reported nicotine vaping, 50% reported low harm for the fetus and 33.3% reported perceiving moderate harm, whereas 18.2% reported high harm. Among women who reported both cannabis and nicotine vaping when asked about cannabis, 63.6% reported low harm, 27.3% reported moderate harm and 9.1% reported high harm, respectively. When asked about nicotine, 45.5% reported low harm, 36.4% moderate harm and 18.2% reported high harm.

Among women who were postpartum and reported vaping cannabis, 87.5% reported perceiving low harm for their child, 12.5% reported moderate harm and no one reported high harm. Among those who were postpartum and reported vaping nicotine, 52.4% reported low harm, 28.6% reported moderate harm and 19% reported high harm. Among women who were postpartum and reported both cannabis and nicotine vaping, when asked about cannabis, 50% reported low harm, 8.3% reported moderate harm and 41.7% reported high harm. When asked about nicotine, 58.3% reported low harm, 8.3% moderate harm and 33.3% reported high harm, as shown in Table 5.

### 3.3. Beliefs About Vaping

Among women who reported cannabis vaping, 22.6% indicated they believed the general public disapproved, while 64.5% reported mixed opinions, whereas 12.9% reported they thought the general public approved. Among those who reported nicotine vaping 38.6% expressed a perception of disapproval, while 49.1% reported mixed opinions, and 12.3% believed the general public approved. Among women who reported using both substances, 30.4% perceived disapproval, while 65.2% reported experiencing mixed opinions, and 4.3% believed there was approval.

Among women who reported cannabis vaping, 12.9% indicated they perceived disapproval from those significant to them, while 51.6% perceived mixed opinions, and 35.5% perceived approval. Among women who reported nicotine vaping, 29.8% perceived disapproval, 43.9% perceived mixed opinions, and 26.3% perceived approval. Among women who reported vaping both cannabis and nicotine, 8.7% perceived disapproval, while 65.2% perceived mixed opinions, and 26.1% perceived approval from people important to them.

Among women who reported cannabis vaping, 25.8% reported they thought people who were important to them disapproved, 41.9% perceived mixed opinions and 32.3% perceived approval. Among women who reported nicotine vaping, 56.1% perceived disapproval, 28.1% perceived mixed opinions, and 15.8% perceived approval. Among women who vaped both cannabis and nicotine, 34.8% perceived disapproval, 52.2% perceived mixed opinions and 13% perceived approval.

Regarding the general public’s beliefs about vaping during pregnancy and postpartum among women who reported cannabis use, 71% indicated they believed the general public disapproved, while 29% reported mixed opinions and no one thought the general public approved. Among those who reported nicotine use, 87.7% expressed a perception of disapproval, while 8.8% reported mixed opinions, and 3.5% believed the general public approved. Among women who reported using both substances, 87% perceived disapproval, while 13% reported experiencing mixed opinions, and no one believed there was approval, as shown in Table 6.

### 3.4. Reasons Women Consulted a Healthcare Provider

The most frequent reasons women consulted their HCPs were potential harm during pregnancy (45%), fetus’ health (42.3%) and their child’s health (28.8%), as shown in Table 7.

## 4. Discussion

This study examined women’s vaping patterns and perceptions during pregnancy and postpartum. In our sample, nicotine was most commonly vaped by the participants followed by cannabis vaping and then both nicotine and cannabis vaping. Among the women who chose cannabis or cannabis and nicotine, 50% reported vaping THC, while 42.6% used a combination of THC and CBD, and 3.7% vaped CBD exclusively. In terms of frequency, the majority of women (64.9%) reported daily vaping, with 15.3% displaying an inconsistent vaping pattern. A smaller proportion vaped 1–2 days per week (9.9%), and 9% vaped 5–6 days per week.

Our findings suggest that flavor preferences are common among women who vape, with fruit flavors being particularly dominant, followed by menthol or mint and candy, desserts or sweets. These findings align with data from the Canadian Tobacco and Nicotine Survey (2021) that found that fruity flavours are preferred, followed by mint and menthol [34]. Flavors, particularly fruit flavors, are well-known favorites for initiating vaping among women and youth [35]. For pregnant women, flavors may be especially important as they can help mitigate nausea and provide a smoother vaping experience. Pregnant women experience heightened sensitivity to bitter tastes, and increased nausea, which may influence their preferences for flavored products, including menthol cigarettes and other novel options [36,37,38].

We found that women commonly turn to vaping as a means of managing various mental health challenges, with anxiety being the most frequently cited reason, followed by depression and post-traumatic stress disorder (PTSD). Many women reported that vaping helped them relax or manage anxiety and depression, particularly during pregnancy or the postpartum period, highlighting the potential role of vaping in coping with stress during vulnerable life stages. The perception of lower harm compared to smoking was frequently mentioned as motivating factors for choosing to vape nicotine. The physical symptoms that led women to vape were also diverse, with sleep disturbances being the most common, followed by headaches, lack of appetite, nausea, and pain. These findings underscore the complex and multifaceted reasons women may turn to vaping during pregnancy and postpartum and are consistent with other studies. For example, these findings align with studies conducted in the United States, where pregnant women reported using cannabis to manage stress, anxiety, chronic pain, nausea, and vomiting [39,40]. Similarly, a qualitative study in Canada found that women often reframed their cannabis use as a form of symptom management, reflecting a nuanced understanding of the perceived risks and benefits. The authors of the Canadian study also suggested that this shift may be driven, in part, by a desire to mitigate the stigma associated with cannabis use during pregnancy by presenting it as a therapeutic practice [15,41].

In a different phase of this study, we conducted semi-structured interviews with 22 of the 111 women who participated in the survey [42]. Those who used nicotine primarily viewed vaping as a means of reducing the harm associated with smoking traditional tobacco cigarettes. In contrast, women who vaped cannabis often perceived it as a therapeutic tool, beneficial for both mental and physical health. They also tended to view cannabis as less harmful than nicotine, alcohol, or other substances, including prescription medications [42]. This distinction underscores the different motivations and health perceptions that guide women’s choices to vape cannabis or nicotine, highlighting the complex ways in which substances are framed in relation to their potential risks and benefits. For example, vaping nicotine is preferred to smoking in order to reduce potential harms related to women’s health as well as the health of their babies and others around them [10].

Our study highlights differences in women’s perceptions of harm associated with cannabis and nicotine use including among those who used both cannabis and nicotine. While the majority viewed cannabis vaping as low harm, perceptions of nicotine use were more evenly distributed across low, moderate, and high harm categories. Among pregnant women, perceptions of fetal harm varied notably between cannabis and nicotine, with a higher proportion considering nicotine use as highly harmful. This aligns with broader trends in which cannabis is generally perceived as less harmful than tobacco products [43]. The legalization of recreational cannabis in Canada and its association with a more natural product may contribute to this perception. Given the conflicting information available, women often struggle to navigate the choice of vaping during pregnancy and postpartum. Qualitative interviews have revealed that women seek out research and rely heavily on the experiences of others, including other women, to guide their decisions [42].

This study revealed diverse perceptions of societal attitudes toward vaping among women who use both cannabis and nicotine, with many reporting mixed opinions and significant variation in perceived approval or disapproval from both the general public and important individuals in their lives. During pregnancy and the postpartum period, women felt a stronger sense of disapproval, particularly regarding nicotine use, suggesting that societal norms and support networks might exert considerable influence during these critical stages.

The most frequent reasons women consulted their healthcare providers regarding their nicotine or cannabis vaping were concerns about potential harm to their pregnancy, the health of their fetus, and the well-being of their child. This suggests that women are deeply concerned about the impact of their behaviors on their babies and child health and seek professional guidance to better understand the risks involved. These concerns likely reflect a broader desire to make informed decisions during pregnancy as other studies also found [44,45]. These findings highlight the importance of HCPs addressing these topics in a non-judgmental and supportive manner. It also emphasizes the need for clear, evidence-informed information about the effects of cannabis or nicotine vaping on maternal and fetal health, as well as the role of HCPs in helping women navigate these risks. To support healthcare providers in initiating conversations with women about vaping and harm reduction, we developed a resource in response to women citing healthcare providers’ discomfort or lack of confidence in discussing vaping during pregnancy and the postpartum period [46]. The resource provides context on vaping, explores why women vape during pregnancy and postpartum, and offers guidance on how HCPs can begin conversations with women about vaping and harm reduction [46].

This study has several limitations. First, the sample was recruited through social media, and thus it may not be representative of the broader population. As with other studies, the participants were predominantly White [15] and their views and patterns may not represent other racial/ethnic groups. Additionally, our findings may not be generalizable to pregnant and postpartum women who vape cannabis and/or nicotine in other countries, particularly those where cannabis is not legal as this study was conducted in Canada, where cannabis use is legal.

## 5. Conclusions

This study sheds light on women’s vaping behaviors during pregnancy and the postpartum period, revealing that nicotine vaping was the most common, followed by cannabis and dual-use vaping. Women reported vaping as a means of managing mental health challenges, such as anxiety and depression, as well as physical symptoms such as nausea and sleep disturbances. Flavor preferences, especially fruit flavors, were prevalent. Perceptions of harm differed between nicotine and cannabis, with cannabis generally viewed as lower risk, particularly for the fetus. Women also expressed concerns about societal disapproval, especially regarding nicotine use, and sought guidance from healthcare providers, emphasizing the need for clear, evidence-based communication. These findings highlight the complexity of vaping decisions during pregnancy and postpartum and underscore the importance of providing women with non-judgmental support and accurate information to help them navigate the potential risks.

## Figures and Tables

**Table 1:** Socio-demographic characteristics of a sample of women who vaped cannabis, nicotine, or both cannabis and nicotine during pregnancy and postpartum.

	N	%
**Age**		
16–19	14	12.6
20–25	49	44.1
26–30	22	19.8
31–35	21	18.9
>36	5	4.5
**Race/ethnicity**		
Black	6	5.4
Indigenous (First Nations, Inuit, or Métis)	8	7.2
Japanese	1	0.9
Latin American	2	1.8
South Asian (e.g., East Indian, Pakistani, Sri Lankan)	1	0.9
Southeast Asian (e.g., Vietnamese, Cambodian, Malaysian, Laotian)	2	1.8
White	84	75.7
Other	4	3.6
I do not know	2	1.8
Refused to answer	1	0.9
**Education**		
Grade school/some high school	10	9.0
Completed high school	32	28.8
Technical/trade school or community college	31	27.9
Some university, no degree	15	13.5
Completed university degree	15	13.5
Post-graduate degree	7	6.3
Refused to answer	1	0.9
**Income**		
CAD 29,999 and under	18	16.2
CAD 30,000–59,999	34	30.6
CAD 60,000–99,999	30	27.0
CAD 100,000 and over	18	16.2
Do not know	5	4.5
Refused to answer	6	5.4
**Marital status**		
Common law	52	46.8
Married	25	22.5
Divorced or separated	4	3.6
Single, never married	28	25.2
Refuse to answer	2	1.8
**Sexual orientation**		
Bisexual	28	25.2
Heterosexual	69	62.2
Homosexual	1	
Other	1	0.9
I do not know	4	3.6
Refused to answer	8	7.2

**Table 2:** Reasons for vaping nicotine, cannabis and both cannabis and nicotine.

	Nicotine (*n* = 57)	Cannabis (*n* = 31)	Cannabis and Nicotine (*n* = 23)	Total
I enjoy vaping.	39 (68.4%)	26 (83.9%)	20 (87.0%)	85 (76.6%)
I can hide vaping more easily than smoking.	29 (50.9%)	17 (54.8%)	16 (69.6%)	62 (55.9%)
I can vape in places where I cannot smoke.	33 (57.9%)	15 (48.4%)	14 (60.9%)	62 (55.9%)
Vaping might help me to relax.	54 (94.7%)	28 (90.3%)	21 (91.3%)	103 (92.8%)
I enjoy vape flavours.	37 (64.9%)	8 (25.8%)	18 (78.3%)	63 (56.8%)
A friend or family member suggested vaping.	12 (21.05%)	4 (12.9%)	7 (30.4%)	23 (20.7%)
Vaping is less harmful to me than smoking.	45 (78.9%)	25 (80.6%)	14 (60.9%)	84 (75.7%)
Vaping is less harmful than smoking to other people around me.	43 (75.4%)	23 (74.2%)	14 (60.9%)	80 (72.1%)
Vaping is less harmful than smoking to my fetus (only for pregnant women).	26 (72.2%)	21 (91.3%)	5 (45.4%)	52 (74.3%)
Vaping is less harmful than smoking to my baby (for postpartum women).	15 (71.43%)	5 (62.5%)	7 (58.3%)	27 (65.8%)
Vaping is more acceptable than smoking to people around me.	43 (75.4%)	24 (77.4%)	16 (69.6%)	83 (74.8%)
I save money by vaping instead of smoking.	37 (64.9%)	11 (35.5%)	13 (56.5%)	61 (55.0%)
Vaping nicotine/cannabis helps me control my appetite and/or weight.	13 (22.8%)	11 (35.5%)	12 (52.2%)	36 (32.4%)
Vaping helps me cut down on the number of cigarettes I smoke.	34 (59.6%)	4 (12.9%)	17 (73.9%)	55 (49.5%)
Vaping might help me stop smoking.	37 (64.9%)	7 (22.6%)	18 (78.3%)	62 (55.9%)
Vaping cannabis is natural and safe.	N/A	23 (74.2%)	3 (13.0%)	26 (23.4%)
Vaping cannabis is not addictive.	N/A	15 (48.4%)	1 (4.3%)	16 (14.4%)
Vaping during pregnancy/postpartum helps me manage anxiety/depression.	45 (78.9%)	28 (90.3%)	22 (95.6%)	95 (85.6%)
Vaping cannabis during pregnancy helps me manage morning sickness.	N/A	20 (64.5%)	(*)	20 (18.0%)
Vaping helps me manage sleep issues.	23 (40.3%)	29 (93.5%)	19 (82.6%)	71 (64%)
Other reasons	7 (12.3%)	9 (29.0%)	5 (21.7%)	21 (18.9%)

Note. Respondents could answer “yes” or “no” and could select all options that applied, regardless of their primary reason for vaping. N/A is not applicable. * This question was not asked of those who vaped cannabis and nicotine.

**Table 3:** Have you ever vaped to improve or manage any of the following?

	%
Anxiety (including phobia, obsessive–compulsive disorder or a panic disorder)	92 (82.9%)
Depression (including dysthymia)	70 (63.1%)
Post-traumatic stress disorder (PTSD) or traumatic event (e.g., abuse or loss)	48 (43.2%)
Mental health disorders such as bipolar disorder, psychosis or schizophrenia	23 (20.7%)
Alcohol or other drug use	25 (22.5%)
Eating disorder	18 (16.2%)
ADD (attention deficit disorder)/ADHD (attention deficit hyperactivity disorder)	22 (19.8%)
Relationship violence	10 (9.0%)
Other significant emotional or mental health problems (please specify)	5 (4.5%)
I have never vaped to manage any of the above	9 (8.1%)
Do not know	2 (1.8%)

Note. Participants could select all that applied.

**Table 4:** Have you ever vaped to improve or manage physical symptoms?

	%
Headaches/migraines	46 (41.4%)
Pain (including arthritis, neuropathy or PMS)	32 (28.8%)
Nausea/vomiting or chemotherapy symptoms	33 (29.7%)
Lack of appetite	41 (36.9%)
Seizures	0 (0%)
Muscle spasms	3 (2.7%)
To shrink tumors or treat cancer	0 (0%)
Problems sleeping	49 (44.1%)
Digestion/gastrointestinal issues (Crohn’s Disease, colitis, IBS, IBD, etc.)	13 (11.7%)
Fibromyalgia	2 (1.8%)
Other condition(s) (please specify):	1 (0.9%)
I have never vaped to treat or improve symptoms	29 (26.1%)
Do not know	11 (9.9%)

Note. Participants could select all that applied.

**Table 5:** How do you think cannabis vaping and cannabis and nicotine vaping affect your health, your fetus, or your child?

	Nicotine (*n* = 57)	Cannabis (*n* = 31)	Cannabis and Nicotine (*n* = 23) When Asked About Cannabis	Cannabis and Nicotine (*n* = 23) When Asked About Nicotine
Health (*n* = 111)				
Low harm	21 (36.8%)	25 (80.6%)	15 (65.2%)	9 (39.1%)
Moderate harm	31 (54.4%)	6 (19.4%)	8 (34.8%)	9 (39.1%)
High harm	5 (8.8%)	0	0	5 (21.7%)
Fetus (*n* = 70)	*n* = 36	*n* = 23	*n* = 11	*n* = 11
Low harm	18 (50.0%)	20 (87.0%)	7 (63.6%)	5 (45.5%)
Moderate harm	12 (33.3%)	2 (8.7%)	3 (27.3%)	4 (36.4%)
High harm	6 (16.7%)	1 (4.3%)	1 (9.1%)	2 (18.2%)
Child (*n* = 41)	*n* = 21	*n* = 8	*n* = 12	*n* = 12
Low harm	11 (52.4%)	7 (87.5%)	6 (50%)	7 (58.3%)
Moderate harm	6 (28.6%)	1 (12.5%)	1 (8.3%)	1 (8.3%)
High harm	4 (19.0%)	0	2 (41.7%)	4 (33.3%)

Note. Column percentage reported. For the purposes of data analysis, these questions were recoded to create a more manageable and interpretable scale. The responses “Extremely harmful” and “Very harmful” were combined into a single category, “High harm”. The response “Moderately harmful” was kept as a distinct category, “Moderate harm”. Finally, the categories “Slightly harmful” and “Not at all harmful” were grouped together into the “Low harm” category.

**Table 6:** Beliefs about vaping from important people and general public.

	Nicotine (*n* = 57)	Cannabis (*n* = 31)	Cannabis and Nicotine (*n* = 23)	Total
General public’s attitudes towards vaping				
Disapproval	22 (38.6%)	7 (22.6%)	7 (30.4%)	36 (32.4%)
Mixed opinions	28 (49.1%)	20 (64.5%)	15 (65.2%)	63 (56.8%)
Approval	7 (12.3%)	4 (12.9%)	1 (4.3%)	12 (10.8%)
The attitudes towards vaping of people who are important to you				
Disapproval	17 (29.8%)	4 (12.9%)	2 (8.7%)	23 (20.7%)
Mixed opinions	25 (43.9%)	16 (51.6%)	15 (65.2%)	56 (50.5%)
Approval	15 (26.3%)	11 (35.5%)	6 (26.1%)	32 (28.8%)
Thoughts of important people about vaping during pregnancy or postpartum				
Disapproval	32 (56.1%)	8 (25.8%)	8 (34.8%)	48 (43.2%)
Mixed opinions	16 (28.1%)	13 (41.9%)	12 (52.2%)	36.9 (41%)
Approval	9 (15.8%)	10 (32.3%)	3 (13%)	22 (19.8%)
General public’s attitudes towards vaping during pregnancy and postpartum				
Disapproval	50 (87.7%)	22 (71%)	20 (87%)	92 (82.9%)
Mixed opinions	5 (8.8%)	9 (29%)	3 (13%)	17 (15.3%)
Approval	2 (3.5%)	0 (0%)	0 (0%)	2 (1.8%)

**Table 7:** Have you ever consulted a healthcare provider about vaping?

	%
Your fertility	10 (9.0%)
Your partner’s fertility	6 (5.4%)
Your health	26 (23.4%)
Other people’s health	9 (8.1%)
Your fetus’ health	47 (42.3%)
Your child’s health	32 (28.8%)
Potential harm during pregnancy	50 (45%)
Potential harm of second- or third-hand vaping exposure during pregnancy	29 (26.1%)
Exposure of infant to vapour postpartum	27 (24.3%)
Treatments to quit vaping	20 (18%)
Benefits of vaping	27 (24.3%)
Potential harms of breastfeeding while vaping	21 (18.9%)
Testing for cannabis/nicotine use in pregnant women	6 (5.4%)
Testing for cannabis/nicotine use in fetus	8 (7.2%)
Testing for cannabis/nicotine use in infants	7 (6.3%)
Other (please specify)	1 (0.9%)

Note. Respondents could answer “yes” or “no”.

## Data Availability

The original contributions presented in this study are included in the article.

## Supplementary Materials

The following supporting information can be downloaded at https://www.mdpi.com/article/10.3390/healthcare13030223/s1: Table S1: Survey questions for women who vape(d) nicotine and/or cannabis in pregnancy and/or postpartum.
