# Supplementary material for: Women’s Experiences with Nicotine and Cannabis Vaping During Pregnancy and Postpartum
Source: Healthcare (Basel). 2025 Jan 22;13(3):223. doi: 10.3390/healthcare13030223 (PMC11817223; doi:10.3390/healthcare13030223)
Supplement: Supplementary file 1 [file healthcare-13-00223-s001.zip › healthcare-3368325-supplementary.pdf]

Table S1. Survey questions for women who vape(d) nicotine and/or cannabis in pregnancy and/or postpartum

1. Are you currently pregnant?
- **Yes**
- **No**

2. If YES, when did you become pregnant? Please provide the date.

3. Did you deliver a baby less than two years ago?
- **Yes**
- **No (end of survey if did not deliver a baby less than two years ago & not currently pregnant)**

4. If YES to Q3, please provide the birthdate.

5. Which of the following statements describe your situation?
- I planned to get pregnant
- I wanted to get pregnant, but not at that time
- I did not want to get pregnant

6. Have you ever vaped, even one time?
- Yes
- No

7. What did/do you usually vape?
- Cannabis
- Nicotine
- Herbs or blends of essential oil (end of survey)
- Cannabis and nicotine

8. Which of the following answers describe your situation? for both
- I vaped cannabis while I was pregnant.
- I vaped nicotine while I was pregnant.
- I vaped both cannabis and nicotine during pregnancy.
- I used to vape nicotine before I was pregnant but stopped when I found out I was pregnant.
- I used to vape cannabis before I was pregnant but stopped when I found out I was pregnant.
- I did not vape when I was pregnant.

9. Did your vaping patterns change during pregnancy?
- Yes, I used to vape cannabis before pregnancy and switched to nicotine during pregnancy.
- Yes, I used to vape nicotine before pregnancy and switched to cannabis during pregnancy.
- Yes, I used to vape nicotine before pregnancy and started vaping cannabis as well during pregnancy.
- Yes, I used to vape cannabis before pregnancy and started vaping nicotine as well during pregnancy.
- No, I kept the same patterns as before pregnancy and vaped cannabis.
- No, I kept the same patterns as before pregnancy and vaped nicotine.
- No, I kept the same patterns as before pregnancy and vaped both nicotine and cannabis.

10. Which of the following answers describe your situation?
- I did not vape during pregnancy but started again after I delivered the baby.
- I vaped during pregnancy and now during postpartum.
- I did not vape during pregnancy or now (end of survey).

11. If you vape cannabis: What do you usually vape?
- THC
- CBD
- Both THC and CBD
- Don’t know

12. How often do you CURRENTLY vape?
- Daily
- Less than daily, but at least once a week

- Less than weekly, but at least once a month
- Less than once a month, but occasionally
- Not at all

13. In a typical week, on how many days do you vape?

- 1-2 days a week
- 3-4 days a week
- 5-6 days a week
- Everyday
- Can't say, there's no consistent pattern

14. Which of the following answers describe your situation? (only for pregnant women)

- I currently vape more than before pregnancy.
- I currently vape less than before pregnancy.
- I currently vape the same as before pregnancy.

15. Which of the following answers describe your situation? (only for postpartum women)

- I currently vape more than when I started, immediately after pregnancy.
- I currently vape less than when I started, immediately after pregnancy.
- I currently vape the same as when I started, immediately after pregnancy.

16. Did your vaping patterns change during the pandemic?

- Yes, I vaped more.
- Yes, I vaped less.
- No, there were no changes in my vaping patterns.

17. What brand do you use for your vaping device? (open question)

18. How did you get your vaping device?

- From a friend
- From a relative
- From my partner
- Free sample
- I bought it
- Other (please specify)

19. Which of the following flavours of e-liquid have you used?

- Tobacco flavour
- Mix of tobacco and menthol
- Menthol or mint
- Fruit flavour
- Candy, desserts, sweets
- Chocolate
- Clove or other spice
- Coffee
- Non-alcoholic drink
- An alcoholic drink
- Unflavoured e-liquid
- Other (please specify)
- I do not use e-liquid

KNOWLEDGE OF HEALTH EFFECTS

1. How do you think vaping nicotine affects your health?

- Not at all harmful
- Slightly harmful

- Moderately harmful
- Very harmful
- Extremely harmful

2. How do you think vaping cannabis affects your health?

- Not at all harmful
- Slightly harmful
- Moderately harmful
- Very harmful
- Extremely harmful

3. How do you think vaping nicotine affects the fetus/child?

- Not at all harmful
- Slightly harmful
- Moderately harmful
- Very harmful
- Extremely harmful

4. How do you think vaping cannabis affects the fetus/child?

- Not at all harmful
- Slightly harmful
- Moderately harmful
- Very harmful
- Extremely harmful

5. Do you enjoy vaping?

- Not at all
- Slightly
- Moderately
- Very much
- Extremely

BELIEFS ABOUT VAPING

1. What do people who are important to you think about vaping?

- All or nearly all approve
- Most approve
- About half approve and half disapprove
- Most disapprove
- All or nearly all disapprove

2. While you were pregnant and/or postpartum, what did people who are important to you say about your vaping?

- All or nearly all approve
- Most approve
- About half approve and half disapprove
- Most disapprove
- All or nearly all disapprove

3. What do you think the general public`s attitude is towards vaping?

- All or nearly all approve
- Most approve
- About half approve and half disapprove
- Most disapprove
- All or nearly all disapprove

4. What do you think the general public`s attitude is towards vaping in pregnant/postpartum women?

- All or nearly all approve
- Most approve
- About half approve and half disapprove
- Most disapprove

- All or nearly all disapprove

REASONS TO VAPE

Which of the following are reasons that you vape? All are YES/NO questions.

- I enjoy vaping.
- I can hide vaping more easily than smoking.
- I can vape in places where I can´t smoke.
- Vaping helps me to relax.
- I enjoy vape flavours.
- A friend or family member suggested vaping.
- Vaping nicotine is less harmful to me than smoking.
- Vaping nicotine is less harmful than smoking to other people around me.
- Vaping nicotine is less harmful than smoking to my fetus (only for pregnant women).
- Vaping nicotine is less harmful than smoking to my baby (for postpartum women).
- Vaping nicotine is more acceptable than smoking to people around me.
- I save money by vaping nicotine instead of smoking.
- Vaping nicotine helps me control my appetite and/or weight.
- Vaping nicotine helps me cut down on the number of cigarettes I smoke.
- Vaping nicotine might help me stop smoking.
- Vaping nicotine during pregnancy/postpartum helps me manage anxiety/depression.
- Vaping nicotine helped me manage sleep issues while pregnant/during postpartum.
- Vaping cannabis is less harmful to me than smoking.
- Vaping cannabis is less harmful than smoking to other people around me.
- Vaping cannabis is less harmful than smoking to my fetus (only for pregnant women).
- Vaping cannabis is less harmful than smoking to my baby (for postpartum women).
- Vaping cannabis is more acceptable than smoking to people around me.
- I save money by vaping cannabis instead of smoking.
- Vaping cannabis helps me control my appetite and/or weight.
- Vaping cannabis helps me cut down on the number of cigarettes I smoke.
- Vaping cannabis might help me stop smoking.
- Vaping cannabis is natural and safe.
- Vaping cannabis is not addictive.
- Vaping cannabis during pregnancy/postpartum helps me manage anxiety/depression.
- Vaping cannabis during pregnancy helps me manage morning sickness.
- Vaping cannabis helps me manage sleep issues.
- Vaping cannabis helped me manage sleep issues while pregnant/during postpartum.
- Other (please specify)

REASONS TO CONSULT A HEALTHCARE PROVIDER

1. Have you ever consulted a health care provider about vaping and: (yes/no questions)

- Your fertility
- Your partner's fertility
- Your health
- Other people´s health
- Your fetus' health
- Your child's health
- Potential harms during pregnancy
- Potential harms of second- or third-hand vaping exposure during pregnancy
- Exposure of infant to vapour post partum
- Treatments to quit vaping
- Benefits of vaping
- Potential harms of breastfeeding while vaping
- Testing for cannabis/nicotine use in pregnant women
- Testing for cannabis/nicotine use in fetus
- Testing for cannabis/nicotine use in infants

- Other (please specify)

2. Have you ever vaped to improve or manage any of the following (select all that apply):

- Anxiety (including phobia, obsessive-compulsive disorder or a panic disorder)
- Depression (including dysthymia)
- Post-traumatic stress disorder (PTSD) or traumatic event (e.g., abuse or loss)
- Mental health disorders such as Bipolar disorder, Psychosis or Schizophrenia
- Alcohol or other drug use
- Eating disorder
- ADD (attention deficit disorder)/ADHD (attention deficit hyperactivity disorder)
- Relationship violence
- Other significant emotional or mental health problem (please specify):_________
- I have never vaped to manage any of the above
- Don't know

3. Have you ever vaped to improve or manage symptoms for any of the following (select all that apply):

- Headaches/migraines
- Pain (including arthritis, neuropathy or PMS)
- Nausea/vomiting or chemotherapy symptoms
- Lack of appetite
- Seizures
- Muscle spasms
- To shrink tumors or treat cancer
- Problems sleeping
- Digestion/gastrointestinal issues (Crohn's Disease, colitis, IBS, IBD, etc.)
- Fibromyalgia
- Other condition(s) (please specify):__________
- I have never vaped to treat or improve symptoms
- Don't know

SOCIO-DEMOGRAPHIC CHARACTERISTICS

1. What is your age? (open question)

2. What is the highest level of formal education that you have completed?

- Grade school/ some high school
- Completed high school
- Technical/ trade school or community college
- Some university, no degree
- Completed university degree
- Post-graduate degree
- I don't know
- Refuse to answer

3. What is the gross income of the people in your household?

- Under $10,000
- $10,000-29,999
- $30,000-44,999
- $45,000-59,999
- $60,000-74,999
- $75,000-99,999
- $100,000-149,999
- $150,000 and over
- I don't know
- Refuse to answer

4. What is your marital status?

- Married
- Common law
- Widowed
- Separated
- Divorced
- Single, never married
- I don’t know
- Refuse to answer

5. How many children do you have? Open question.

6. Which province or territory do you live in?

- Newfoundland and Labrador
- Prince Edward Island
- Nova Scotia
- New Brunswick
- Quebec
- Ontario
- Manitoba
- Saskatchewan
- Alberta
- British Columbia
- Yukon
- Northwest Territories
- Nunavut
- I don’t know
- Refuse to answer

7. Are you First Nations, Métis or Inuk (Inuit)?

- First Nations
- Métis
- Inuk (Inuit)
- Indigenous (other)
- No
- I don’t know
- Refuse to answer

8. Are you now, or have you ever been a landed immigrant in Canada?

- Yes
- No
- I don’t know
- Refuse to answer

9. In what year did you first become a landed immigrant in Canada? (open question and only for those who answered YES)

10. You may belong to one or more racial or cultural groups on the following list. Are you... ?

- White
- South Asian (e.g., East Indian, Pakistani, Sri Lankan)
- Chinese
- Black

- Filipino
- Latin American
- Arab
- Southeast Asian (e.g., Vietnamese, Cambodian, Malaysian, Laotian)
- West Asian (e.g., Iranian, Afghan)
- Korean
- Japanese
- Indigenous (First Nations, Inuit, or Métis)
- Other (please specify)
- I don’t know
- Refuse to answer

11. What is your sexual orientation?

- Heterosexual
- Homosexual
- Bisexual
- Other (please specify)
- I don’t know
- Refuse to answer
